# Triaging women with human papillomavirus infection and normal cytology or low‐grade dyskaryosis: evidence from 10‐year follow up of the ARTISTIC trial cohort

**DOI:** 10.1111/1471-0528.15957

**Published:** 2019-11-07

**Authors:** C Gilham, A Sargent, J Peto

**Affiliations:** ^1^ London School of Hygiene & Tropical Medicine London UK; ^2^ Clinical Virology Manchester University NHS Foundation Trust Manchester UK

**Keywords:** Cervical cancer, cervical screening, cervical intrapepithelial neoplasia grade 3, cytology, human papillomavirus, triage

## Abstract

**Objectives:**

To estimate long‐term cervical intraepithelial neoplasia grade 3 (CIN3) risks associated with different triage strategies for human papillomavirus positive (HPV+) women with a view to reducing unnecessary referrals.

**Design:**

The ARTISTIC trial cohort was recruited in Manchester in 2001–03 and was followed up for CIN3 and cancer notification through national registration until December 2015.

**Results:**

The 10‐year cumulative risk of CIN3+ was much higher for women with HPV16/18 infection (19.4%, 95% CI 15.8–23.8% with borderline/low‐grade cytology and 10.7%, 95% CI 8.3–13.9% with normal cytology) than for those with other HPV types (7.3%, 95% CI 5.4–9.7% with borderline/low‐grade cytology and 3.2%, 95% CI 2.2–4.5% with normal cytology). Among the 379 women with normal to low‐grade cytology and new HPV infection, the 10‐year cumulative CIN3+ risk was 2.9% (95% CI 1.6–5.2%).

**Conclusions:**

The CIN3 risk is confined to women with persistent type‐specific HPV so partial genotyping test assays identifying HPV16/18 as a minimum are essential for efficient risk stratification. Immediate referral to colposcopy for HPV+ women with borderline or low‐grade cytology and referral after a year if still HPV+ with normal cytology may be unnecessary. Low‐grade lesions can safely be retested to identify those with persistent HPV. Recall intervals of 1 year for HPV16/18 and 2 years for other high‐risk HPVs are justified for women with normal cytology and might also be considered for women with borderline/low‐grade cytology. The minimal risk of invasive cancer that has progressed beyond stage 1A must be weighed against the advantages for patients and the NHS of reducing the number of referrals to colposcopy.

**Tweetable abstract:**

Cervical screening would be better for women and cheaper for the NHS if women with HPV and normal to low‐grade cytology were retested after a year or two when many infections will have cleared.

## Tweetable abstract

Cervical screening would be better for women and cheaper for the NHS if women with HPV and normal to low‐grade cytology were retested after a year or two when many infections will have cleared.

## Introduction

Primary human papillomavirus (HPV) testing with cytology triage of HPV‐positive (HPV+) women has been piloted at several sites in England following publication of the ARTISTIC Trial results over three rounds of HPV screening^1^, ^2^, ^3^ and of the pooled results with other randomised trials showing a reduction in long‐term cervical cancer risk.^4^ (HPV refers throughout to the high‐risk human papillomavirus (HR‐HPV) types associated with cervical cancer.) The NHS Cervical Screening Programme (NHSCSP) is already one of the most successful in the world in preventing cervical cancer,^5^, ^6^ and national roll‐out of primary HPV testing, due to be completed by the end of December 2019, should further increase sensitivity. However, colposcopy referrals will also increase without more efficient triage methods for HPV+ women. Unnecessary referral increases NHS costs and inconvenience to patients,^7^ and over‐treatment of cervical intraepithelial neoplasia (CIN) can compromise later birth outcomes.^8^ The majority of new HPV infections are transient and harmless, and about 70% disappear within a year with slower clearance thereafter.^9^ Triage options to identify those whose CIN3+ risk justifies colposcopy referral include cytology, HPV genotyping and delayed retesting to identify the minority whose infection has persisted.^10^, ^11^ Molecular triage tests such as viral or host DNA methylation are also being evaluated.^12^

In England, women are invited for screening 3‐yearly from age 25 to age 49 years then 5‐yearly from age 50 to 64 years. Women who are HPV+ with borderline or worse cytology are referred to colposcopy.^13^ Cytologically normal women who are HPV+ are recalled for annual cytology and HPV testing and referred to colposcopy if still HPV+ after 24 months.

The NHSCSP must strike an acceptable balance between cancer risk and the cost and patient inconvenience of excessive screening and unnecessary treatment. The current policy of reflex cytology for HPV+ samples and immediate colposcopy for high‐grade cytology provides effective management for these women. Those with high‐grade cytology at entry to ARTISTIC included all ten prevalent cancers.^14^ The contentious issue is how to triage HPV+ women with low‐grade or normal cytology. Acknowledging that there is no such thing as zero risk, Castle et al.^15^ proposed a triage policy based on CIN3 risk in which women are returned to routine screening with <2% risk, recalled earlier than the routine screening interval with 2–10% risk and referred to immediate colposcopy with >10% risk. We have compared these thresholds with estimated CIN3 risks for alternative triage protocols including current NHSCSP policy.

## Methods

Women were recruited to the ARTISTIC trial after attending routine cervical screening in Greater Manchester and randomly allocated in a ratio of 3:1 to have the HPV result revealed and acted upon, or concealed. Liquid‐based cytology samples were collected for cytology and HPV testing, and followed for histopathology between 2001 and 2009. Management of women with abnormal cytology was identical in both arms. Women in the revealed arm with normal cytology who tested HPV+ were invited for repeat HPV testing at 12 months and if still positive chose between immediate colposcopy or repeat HPV testing at 24 months. The study was extended to a third round where women on both arms were managed according to national guidelines. HPV testing was performed with Hybrid Capture 2 (Qiagen, Hilden, Germany) with a cut‐off of relative light units/control of 1 pg/µl. Three HPV‐typing assays were used to genotype HC2‐positive samples over various periods of the trial. Any high‐risk HPV type detected by any of the assays was included in the analysis.^2^, ^3^ To recruit adequate numbers of older women, the minimum age was increased from 20 to 30 years when the recruitment target for women aged 20–29 years had been reached, then to 40 years when there were enough aged 30–39 years.^1^

All participants in the study have been traced through the NHS Central Register to December 2015 for mortality and cancer registration, including CIN3. The cohort was linked to the NHSCSP call–recall database to obtain lifetime cervical screening records, including reasons for ceasing screening (usually age or hysterectomy).

No patients were involved in the development of the research. The work was funded by the NIHR‐HTA and Cancer Research UK.

### Statistical analysis

Women were classified hierarchically into mutually exclusive groups: HPV16 or HPV18, any other HPV without HPV16 or HPV18 (i.e. HPV31, ‐33, ‐35, ‐39, ‐45, ‐51, ‐52, ‐56, ‐58, ‐59 or ‐68), and HPV‐negative (HC2+ with untyped HPV or HC2‐negative). We have shown that the 10‐year CIN3+ risk in women with normal cytology is higher among those with HPV16 (12.4%, 95% CI 9.3–16.5%) than those with HPV18 (6.9%, 95% CI 3.8–12.4%) but these types were grouped because in the UK 80% of invasive cervical cancers are caused by them.^16^ In the ARTISTIC population, HPV16 was about twice as common as HPV18.

Infections detected in the first HPV sample taken after entry were classified as new or persistent by comparing HPV genotypes with those identified at entry. Women with both new and persistent infections were classified as persistent. This analysis was stratified by time from entry to next test, which varied by cytology and randomisation arm: those with normal cytology were invited for a repeat HPV test after 1 year in the revealed arm, whereas in the concealed arm they were invited for routine follow‐up cytology after 3 years. All women with borderline or low‐grade cytology were invited for repeat cytology after 6 months.

Cumulative CIN3+ risks were estimated by Kaplan–Meier methods modified to allow for interval‐censoring. Cumulative risks were calculated from entry (round 1) and also from round 2 (defined as the first HPV test taken 30–48 months after entry). Women were censored at date of last smear before hysterectomy except 608 women who were censored at last smear within the trial as they were not successfully linked to call–recall data. All analyses were censored on 30 April 2015 to allow for late cancer registration. CIN3+ histology was backdated to the beginning of follow up (round 1 or round 2) where the histology/registration occurred within a year of the beginning of follow up. These cases at time zero give an initial step in the Kaplan–Meier curve showing prevalence. Later CIN3+ histology/registration dates were backdated to the first test in the preceding year, then further backdated to the midpoint of the interval between that test and the preceding test to estimate the approximate date when they became screen‐detectable. The cumulative risks therefore include CIN3s which would be diagnosed if screened at that point in time. Cervical cancers are shown in brackets in the tables by time to cancer registration, including those preceded by CIN3 diagnosis. All analyses were programmed in STATA 15.1 (Stata Corp 2017; College Station, TX, USA).

## Results

The analysis includes the 24 496 women who were successfully flagged for cancer incidence and mortality. From enrolment to the end of follow up 482 CIN3 and 23 invasive cervical cancers were diagnosed from national registrations or from local histopathology data. Of the 13 591 women who had an HPV test at round 2 approximately 3 years after entry, 75 were diagnosed with CIN3 and seven with invasive cervical cancer before April 2015. Average follow up was 12 years.

**Figure 1:**
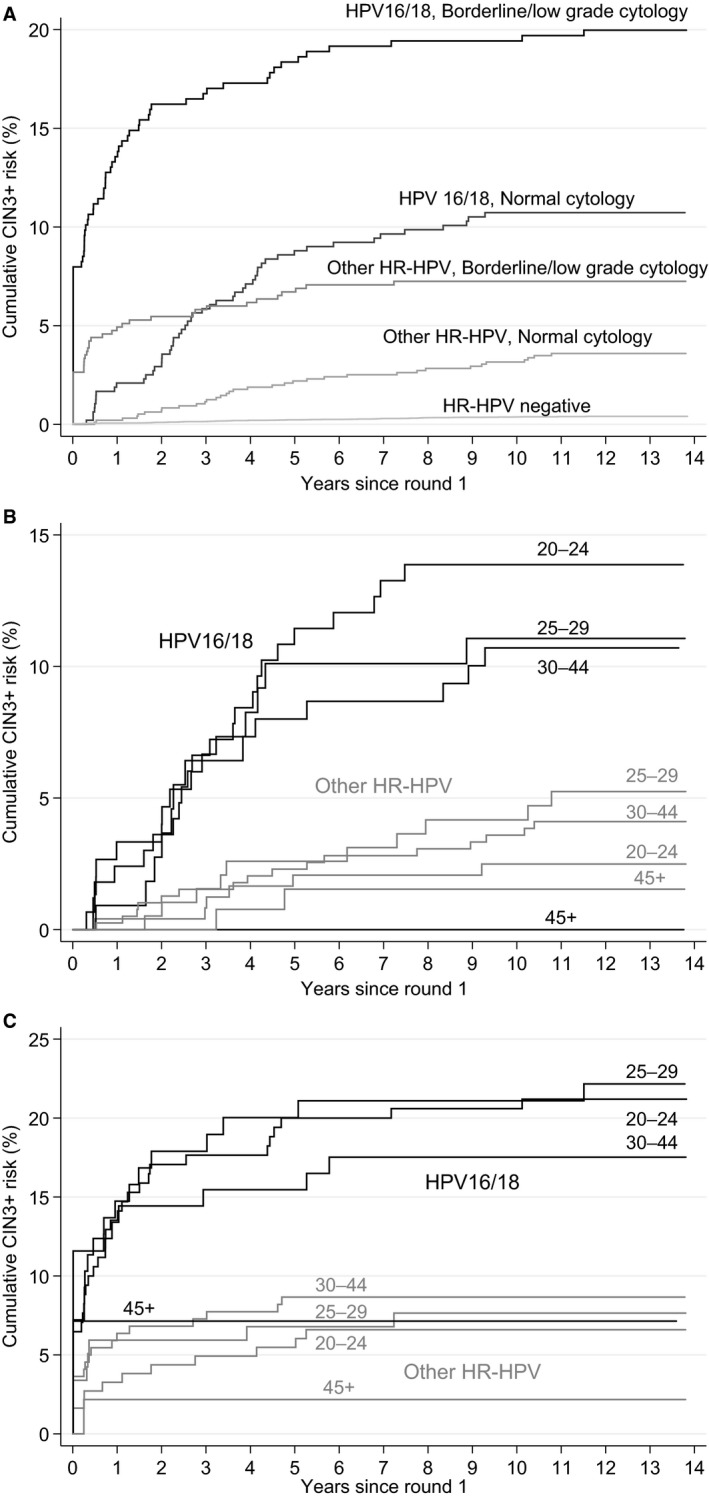
Cumulative CIN3+ risk by HPV type: by cytology (A), by age in women with normal cytology at entry (B), and by age in women with borderline/low‐grade cytology at entry (C).

There were 2383 high‐risk HPV+ women with normal, borderline or low‐grade cytology at entry (Table 1). The majority of this triage population had normal cytology (60% in round 1, 71% in round 2), and roughly equal proportions of the remainder had borderline or low‐grade cytology. The 10‐year cumulative risk of CIN3+ was much higher with HPV16/18 infection (Table 1 and Figure 1A: 19.4%, 95% CI 15.8–23.8% with borderline/low‐grade cytology and 10.7%, 95% CI 8.3–13.9% with normal cytology) than with other HPV types (7.3%, 95% CI 5.4–9.7% with borderline/low‐grade cytology and 3.2%, 95% CI 2.2–4.5% with normal cytology). The higher risk at round 1 in ARTISTIC was largely accounted for by higher prevalence of high‐grade cytology, as the CIN3 risks stratified by cytology were similar in both rounds (see Supplementary material, Table S1). The proportion of HPV+ women who had high‐grade dyskaryosis was 15% aged <45 years and 11% aged 45–64 years in round 1, and only 5% aged <45 years and 2% aged 45–64 years in round 2. Among HPV+ women with normal to low‐grade cytology Figure 1(B and C) shows little effect of age on cumulative CIN3+ risk below age 45 years (*P* = 0.2) but a much lower 10‐year CIN3+ rate (1.6%; 4/243) at age 45 years or over (*P* < 0.0001 compared with women aged under 45 years). Among women with high‐grade cytology at entry there was no age difference (cumulative risks of 55.1% and 53.6% in women aged under and over 45 years, respectively, *P* = 0.9). In women who were HPV+ at entry the cumulative CIN3 and cervical cancer risks showed opposite effects in relation to age. Respective cumulative risks (average follow up 12 years) for ages at entry 20–29, 30–44 and 45–64 years were 16.0% (236/1475), 16.0% (165/1032) and 7.0% (19/273) for CIN3 and 0.2% (3/1475), 1.0% (10/1032) and 1.5% (4/273) for cervical cancer. The ten prevalent invasive cancers were all diagnosed within 7 months of entry, and all presented with high‐grade cytology. In contrast, only two of the 13 incident cervical cancers diagnosed 4 or more years later had abnormal cytology at entry (one borderline, one moderate).

**Table 1:** Cumulative CIN3+ risks from entry by HPV partial genotype and cytology. Invasive cervical cancers (ICC) are also shown in brackets

Follow‐up from entry											
HPV/cytology at entry	*n* (%^a^) at baseline	To Dec 2015^b^	2.5‐year risk from entry			5‐year risk from entry			10‐year risk from entry		
		*n* CIN3+ (ICC)	*n* CIN3+ (ICC)	%	95% CI	*n* CIN3+ (ICC)	%	95% CI	*n* CIN3+ (ICC)	%	95% CI
**HR‐HPV‐negative** c	21716	86 (6)^d^	29 (1)	0.13%	(0.09–0.19%)	50 (2)	0.23%	(0.18–0.30%)	80 (6)	0.37%	(0.30–0.47%)
**HR‐HPV‐positive**	2780	419 (17)	337 (9)	12.1%	(11.0–13.4%)	385 (9)	13.9%	(12.6–15.2%)	413 (12)	14.9%	(13.6–16.3%)
**Normal cytology**											
HPV16/18	478 (17.2%)	51 (2)	23	4.8%	(3.2–7.2%)	42	8.8%	(6.6–11.7%)	51	10.7%	(8.3–13.9%)
Other HR‐HPV	961 (34.6%)	34 (4)	9	0.9%	(0.5–1.8%)	21 (2)	2.2%	(1.4–3.4%)	30 (2)	3.2%	(2.2–4.5%)
All HR‐HPV+	1439 (51.8%)	85 (6)	32	2.2%	(1.6–3.1%)	63 (2)	4.4%	(3.5–5.6%)	81 (2)	5.7%	(4.6–7.0%)
**Borderline/low‐grade cytology**											
HPV16/18	376 (13.5%)	75 (1)	61	16.2%	(12.9–20.4%)	69	18.4%	(14.8–22.7%)	73	19.4%	(15.8–23.8%)
Other HR‐HPV	568 (20.4%)	41	31	5.5%	(3.9–7.7%)	38	6.7%	(4.9–9.1%)	41	7.3%	(5.4–9.7%)
All HR‐HPV+	944 (34.0%)	116 (1)	92	9.8%	(8.0–11.8%)	107	11.4%	(9.5–13.6%)	114	12.1%	(10.2–14.4%)
**Moderate/severe cytology**											
HPV16/18	250 (9.0%)	159 (5)	158 (4)	63.2%	(57.3–69.2%)	159 (4)	63.6%	(57.7–69.5%)	159 (5)	63.6%	(57.7–69.5%)
Other HR‐HPV	147 (5.3%)	59 (4)	55 (4)	37.4%	(30.2–45.8%)	56 (4)	38.1%	(30.8–46.5%)	59 (4)	40.2%	(32.8–48.6%)
All HR‐HPV+	397 (14.3%)	218 (10)^e^	213 (9)^e^	53.7%	(48.8–58.6%)	215 (9)^e^	54.2%	(49.4–59.1%)	218 (10)^e^	54.9%	(50.1–59.9%)
**All women from entry**	24496	505 (23)	366 (10)			435 (11)			493 (18)		

a Percentages are given out of the 2780 total high‐risk (HR) HPV+ women.

b In 12–14 years of follow up.

c HC2‐negative or HC2‐positive with no HR‐HPV detected.

d One invasive cancer diagnosed 4 months after entry and four cancers diagnosed more than 2.5 years after entry tested HC2‐negative, but HR‐HPV was detected on later retesting of the entry sample by polymerase chain reaction, so five of the six cancers shown as HR‐HPV‐negative might have been HPV+ with a modern HPV assay.^14^

e One invasive cancer diagnosed 4 months after severe cytology at entry which tested HC2‐positive with insufficient sample for typing is shown in the table as HR‐HPV+.

Table 2 shows the result of the first HPV test after entry in women who were HPV+ with normal to low‐grade cytology at entry. The 10‐year CIN3+ risk was much lower in women with normal cytology at entry who had cleared their initial infection than in those with persisting infections. The proportion of women with normal cytology who had cleared their initial infection increased from 57% after 1 year to 74% after 3 years and 79% after 5 years, but the proportion acquiring new infections also increased (6% at 1 year, 8% at 3 years, 10% at 5 years). The majority of those with borderline or low‐grade cytology at entry returned for a repeat test after approximately 6 months, when 32% had cleared their initial infection (10‐year CIN3+ risk 2.3%, 95% CI 0.9–5.9% cleared; 17.7%, 95% CI 14.2–21.9% persisting).

**Table 2:** Cumulative CIN3+ risks following next HPV test in women who were high‐risk (HR) HPV+ with normal, borderline or low‐grade cytology at entry. Invasive cervical cancer (ICC) cases are also shown in brackets

Mean time to next HPV test and HPV status	*n* women	*n* CIN3+ (ICC)	2.5‐year risk from next test			5‐year risk from next test			10‐year risk from next test		
			*n* CIN3+ (ICC)	%	95% CI	*n* CIN3+ (ICC)	%	95% CI	*n* CIN3+ (ICC)	%	95% CI
**Normal cytology** b											
*1 year* a											
HPV cleared	323 (51.4%)	7^c^	3	0.9%	(0.3–2.9%)	4	1.2%	(0.5–3.3%)	7	2.2%	(1.1–4.6%)
HPV new infection	37 (5.9%)	0	0	0%		0	0%		0	0%	
HPV persisting	269 (42.8%)	37 (2)	25	9.3%	(6.4–13.4%)	32 (1)	11.9%	(8.6–16.4%)	37 (2)	13.8%	(10.2–18.5%)
HPV16/18 persisting	103 (52.3%)	25 (1)	18	17.5%	(11.4–26.3%)	23	22.3%	(15.4–31.7%)	25 (1)	24.3%	(17.1–33.8%)
Other HR‐HPV persisting	166 (38.4%)	12 (1)	7	4.2%	(2.0–8.6%)	9 (1)	5.4%	(2.9–10.2%)	12 (1)	7.3%	(4.2–12.4%)
*3 years* a											
HPV cleared	193 (66.3%)	1^d^	1	0.5%	(0.1–3.6%)	1	0.5%	(0.07–3.6%)	1	0.5%	(0.07–3.6%)
HPV new infection	22 (7.6%)	1	0	0%		0	0%		1	4.6%	(0.7–28.1%)
HPV persisting	76 (26.1%)	17 (2)	14 (1)	18.4%	(11.4–29.1%)	15 (1)	19.7%	(12.4–30.6%)	17 (2)	22.4%	(14.6–33.5%)
HPV16/18 persisting	31 (27.9%)	6	6	19.4%	(9.2–38.1%)	6	19.4%	(9.2–38.1%)	6	19.4%	(9.2–38.1%)
Other HR‐HPV persisting	45 (25.0%)	11 (2)	8 (1)	17.8%	(9.3–32.4%)	9 (1)	20.0%	(11.0–34.9%)	11 (2)	24.4%	(14.4–39.8%)
*5 years* a											
HPV cleared	101 (69.2%)	1^e^	1	1.0%	(0.1–6.8%)	1	1.0%	(0.1–6.8%)			
HPV new infection	14 (9.6%)	1	1	7.1%	(1.0–40.9%)	1	7.1%	(1.0–40.9%)			
HPV persisting	31 (21.2%)	11	9	29.0%	(16.3–48.4%)	11	35.5%	(21.5–54.9%)			
HPV16/18 persisting	20 (32.8%)	9	8	40.0%	(22.4–64.3%)	9	45.0%	(26.5–68.7%)			
Other HR‐HPV persisting	11 (12.9%)	2	1	9.1%	(1.3–49.2%)	2	18.2%	(4.9–55.3%)			
**Borderline/low‐grade cytology** f											
*6 months* a											
HPV cleared	153 (27.5%)	4	3	2.0%	(0.6–6.0%)	4	2.6%	(1.0–6.8%)	4	2.6%	(1.0–6.8%)
HPV new infection	24 (4.3%)	0	0			0			0		
HPV persisting	379 (68.2%)	67	59	15.6%	(12.3–19.6%)	65	17.2%	(13.7–21.3%)	67	17.7%	(14.2–21.9%)
HPV16/18 persisting	178 (78.8%)	45	39	21.9%	(16.5–28.7%)	43	24.2%	(18.5–31.2%)	45	25.3%	(19.6–21.4%)
Other HR‐HPV persisting	201 (60.9%)	22	20	10.0%	(6.5–15.0%)	22	11.0%	(7.4–16.2%)	22	11.0%	(7.4–16.2%)

a The mean and range follow‐up times were: 1.13 years (<2 years), 3.12 years (2–4 years), 5.19 years (≥4 years) for normal cytology and 6.4 months (<2 years) for borderline or low‐grade cytology.

b 317 women (including 9 CIN3+) with cytology and no HPV at follow up and 56 women with no follow up recorded (no CIN3+) are excluded.

c 1 had same type after an intermediate negative HPV test, 2 cleared and were infected with new HR‐HPV types and 4 cleared and had no subsequent HPV tests before diagnosis (sometimes several years later).

d HR‐HPV not known as sample insufficient for typing.

e Assumed cleared (HC2‐negative) but same type at CIN3 diagnosis as at entry.

f 350 with cytology and no HPV at follow up (39 CIN3+), 27 women with no follow up within 2 years (6 CIN3+) and 11 women with no follow up recorded (no CIN3+) are excluded.

HPV16 was more likely to persist than other high‐risk HPV types. In HPV+ women with normal cytology at entry retested at 1 year, 55% (74/135) of HPV16 infections persisted compared with 39% (195/494) of non‐HPV16 infections (*P* = 0.001). Among women with borderline cytology at entry, 80% (140/174) of HPV16 infections persisted until the next test 6 months later compared with 63% (239/382) of non‐HPV16 infections (*P* < 0.001).

Risks for CIN3+ following round 2 among women who tested HPV‐negative at entry (Table 3) emulate a second round of HPV primary screening. The new infection rate was low: 11.9% in women aged 25–29 years, 4.6% in women aged 30–39 years and 1.6% in women aged 40–64 years. Among the 387 women with a new infection, 70% had normal cytology, 28% had borderline/low‐grade cytology and just 2% had high‐grade cytology. Among the 269 women with normal cytology and the 110 women with borderline/low‐grade cytology, the 10‐year CIN3+ risks were 1.5% (95% CI 0.6–3.9%) and 6.4% (95% CI 3.1–12.9%), respectively. Fewer of these infections persisted compared with those detected at baseline (shown in Table 2): in women with normal cytology, the 12‐month persistence rate was 26% following a new infection at round 2 compared with 43% from any infection detected at baseline; and in women with borderline/low‐grade cytology, the 6‐month persistence rate was 40% following a new infection at round 2 compared with 68% from any infection detected at baseline.

**Table 3:** Cumulative CIN3+ risks following round 2 in women who tested high‐risk (HR) HPV‐negative^c^ at entry. Invasive cervical cancer (ICC) cases are also shown in brackets

HPV and cytology test result at round 2 in women who tested HR‐HPV negative at baseline	*n* (%^a^) at round 2	*n* CIN3+ (ICC)^b^	2.5 year risk from round 2			5 year risk from round 2			10 year risk from round 2		
			*n* CIN3+	%	95% CI	*n* CIN3+ (ICC)	%	95% CI	*n* CIN3+ (ICC)	%	95% CI
**HR‐HPV negative** c	11,945 (96.9%)	16 (2)	8	0.07%	(0.03–0.13%)	11 (1)	0.09%	(0.05–0.17%)	16 (2)	0.14%	(0.08–0.22%)
**HR‐HPV positive**	387 (3.1%)	14 (1)^d^	13 (1)	3.4%	(2.0–5.7%)	14 (1)	3.6%	(2.2–6.0%)	14 (1)	3.6%	(2.2–6.0%)
**Normal cytology**											
HPV16/HPV18	75 (19.4%)	2 (1)^d^	1 (1)	1.3%	(0.2–9.1%)	2 (1)	2.7%	(0.7–10.2%)	2 (1)	2.7%	(0.7–10.2%)
Other HR‐HPV	194 (50.1%)	2	2	1.0%	(0.3–4.1%)	2	1.0%	(0.3–4.1%)	2	1.0%	(0.3–4.1%)
All HR‐HPV+	269 (69.5%)	4	3	1.1%	(0.4–3.4%)	4	1.5%	(0.6–3.9%)	4	1.5%	(0.6–3.9%)
**Borderline/low‐grade cytology**											
HPV16/HPV18	49 (12.7%)	4	4	8.2%	(3.1–20.3%)	4	8.2%	(3.1–20.3%)	4	8.2%	(3.1–20.3%)
Other HR‐HPV	61 (15.8%)	3	3	4.9%	(1.6–14.5%)	3	4.9%	(1.6–14.5%)	3	4.9%	(1.6–14.5%)
All HR‐HPV+	110 (28.4%)	7	7	6.4%	(3.1–12.9%)	7	6.4%	(3.1–12.9%)	7	6.4%	(3.1–12.9%)
**Moderate/Severe cytology**											
HPV16/HPV18	4 (1.0%)	1	1	25.0%	(4.0–87.2%)	1	25.0%	(4.0–87.2%)	1	25.0%	(4.0–87.2%)
Other HR‐HPV	4 (1.0%)	2	2	50.0%	(15.5–94.2%)	2	50.0%	(15.5–94.2%)	2	50.0%	(15.5–94.2%)
All HR‐HPV+	8 (2.1%)	3	3	37.5%	(13.9–77.1%)	3	37.5%	(13.9–77.1%)	3	37.5%	(13.9–77.1%)
**Total women**	12,332^e^	30 (3)	21 (1)			25 (2)			30 (3)		

a Percentages by cytology are given out of 387 HR‐HPV+ women.

b In 12 years of follow up.

c HC2‐negative or HC2‐positive with no HR‐HPV detected.

d HPV16 was detected at round 2 in this prevalent invasive cancer. This woman tested HC2‐negative at entry but HPV16 was detected on later retesting of the entry sample by polymerase chain reaction, so it might have been HPV‐positive with a modern HPV assay.^14^

e 54 women with no cytology taken at round 2 are excluded from the table.

## Discussion

### Main findings

The convention that a CIN3 risk of about 10% is a reasonable threshold for immediate colposcopy^12^, ^15^ would support the current triage strategies adopted by the NHSCSP (Table 4). However, partial genotyping allows further stratification, implying rapid referral only of women with HPV16/18 infection and borderline/low‐grade cytology (5‐year risk 18%). These women constitute 16% of the HPV+ population who do not have high‐grade dyskaryosis (Tables 1 and 4). Over half of the women with borderline/low‐grade cytology had non‐16/18 HPV infections, of which 40% cleared after 6 months. The 5‐year cumulative risk in those persisting beyond 6 months was 11.0% (95% CI 7.4–16.2%) (Tables 2 and 4). This indicates that the triage protocol for those with borderline/low‐grade cytology being rolled out in England is too conservative. There would be a 23% reduction in referrals if those with non‐16/18 HPV types were recalled after 6 months and referred only if persistent, and extending the recall interval to 1 year would further reduce the number of referrals.

**Table 4:** Clinical outcomes of different triage strategies of high‐risk (HR) HPV+ women by cytology and HPV genotype

Strata	Action	Estimated proportion of referrals % (*n*)^a^	5 year cumulative CIN3 risk
**Current policy:**			
Normal cytology	Repeat 12 months, referral if abnormal cytology	NK	NK
	Repeat 12 months, repeat 12 months if still HPV+^b^	49% (306/629)	10.5% (7.5–14.5%)
	Repeat 24 months, referral if still HPV+	33% (97/290)^c^	15.5% (9.6–24.3%)
Borderline/low‐grade cytology	Immediate referral	100%	11.4% (9.5–13.6%)
**Alternative strategies (n and % of all HPV+)**			
Normal HPV16/18			
(*n* = 478, 20%)	Immediate referral	100%	8.8% (6.6–11.7%)
	Repeat 12 months, referral if persistent	52% (103/197)	22.3% (15.4–31.7%)
	Repeat 36 months, referral if persistent	28% (31/111)	19.4% (9.2–38.1%)
Normal other HR‐HPV			
(*n* = 961, 40%)	Immediate referral	100%	2.2% (1.4–3.4%)
	Repeat 12 months, referral if persistent	38% (166/432)	5.4% (2.9–10.2%)
	Repeat 36 months, referral if persistent	25% (45/180)	20.0% (11.0–34.9%)
Borderline/low‐grade HPV16/18			
(*n* = 376, 16%)	Immediate referral	100%	18.4% (14.8–22.7%)
	Repeat 6 months, referral if persistent	79% (178/226)	24.2% (18.5–31.2%)
Borderline/low‐grade other HR‐HPV			
(*n* = 568, 24%)	Immediate referral	100%	6.7% (4.9–9.1%)
	Repeat 6 months, referral if persistent	61% (201/330)	11.0% (7.4–16.2%)

a *n* gives the number used to calculate the estimated proportion of referrals.

b Plus normal cytology.

c Based on average 36‐month recall interval (24–48 months).

Women with normal cytology and HPV16/18 infections persisting for 1 year have a substantial CIN3+ risk and delaying recall for 1 year reduces referrals by around half (Table 4). The cumulative 5‐year risk in women with non‐HPV16/18 infections (2.2%) is near the threshold for routine recall recommended by Castle et al.^15^ so a 2‐year delay in recalling them appears justified. A delay in retesting of a year for HPV16/18 and 2 years for other HPV types for HPV+ women with normal cytology was adopted by some sites during the pilot but will not continue after the national roll‐out of primary HPV testing.

Reflex cytology provides a good triage strategy for women undergoing their first HPV test as well as for previously screened women. However, CIN3+ rates were significantly lower in women with a previous negative HPV test (Table 3). After the first round of HPV screening the HPV‐negative majority (89% in ARTISTIC) have a 10‐year CIN3+ risk of only 0.4%. At the second round of primary HPV testing the great majority of HPV+ women who were HPV‐negative at their previous test will have normal cytology or the low‐grade dyskaryosis associated with a new HPV infection (Table 3). Their 10‐year CIN3+ risk is only 1.5% with normal cytology and 6.4% with borderline or low‐grade cytology, and these women could safely be recalled for repeat testing after at least a year. The HPV+ minority at round 1 have a 10‐year CIN3+ risk of 14.9%, account for 83% of subsequent CIN3+ cases, and remain at much higher risk for invasive cancer beyond 10 years (Table 1; 5/2780 versus 0/21 716 cancers beyond 10 years, *P* < 0.0001). The continuing clinical burden of repeated HPV testing, cytology and colposcopy will therefore be largely confined to women who are HPV+ at their first test, some of whom will have long‐standing pre‐cancer missed by previous cytology.^14^

### Strengths and limitations

We have individually linked HPV, screening and cancer registration data for this large cohort with almost complete follow up over 14 years. The cohort included women being screened at age 20–24 years as well as a large number of women aged 40–64 years. Results at rounds 1 and 2 reflect both the introduction of HPV testing and the much lower CIN3 rate at subsequent rounds. The main limitations are that the HC2 HPV assay was the only commercially available test in 2001 but is no longer used by the NHSCSP, and that colposcopy was rarely performed without abnormal cytology and biopsies were not taken without atypical colposcopic appearance. Further evidence is needed on long‐term cancer risks following negative HPV tests using modern assays to determine whether their sensitivity is adequate (see Table 1 footnotes), and on biopsy of persistent HPV with normal colposcopic appearance.

### Interpretation

#### Genotyping

Some of the approved NHSCSP assays can identify genotypes HPV16/18, but triage depends only on a positive/negative result. Partial genotyping allowing further stratification is beneficial for two reasons. First, the risk of CIN3+ is substantially higher for HPV16/18 than for other HPV types irrespective of cytology (Table 1). The latter constitute the majority of women, their clearance rates are higher, and delaying their repeat test will have the greatest impact on the number of colposcopy referrals. The second advantage is that persistent HPV16 or HPV18 infections can be distinguished from new infections. As the recall interval increases, so does the proportion of double‐positive infections that are in fact one infection clearing followed by a new infection. Such type swaps occurred in 12% (37/307) of women with apparently persistent HPV and normal cytology after 1 year, 22% (22/98) after 3 years and 31% (14/45) at 5 years (Table 2). The 5‐year CIN3 risk for those with type‐specific persistence is four to five times greater than for those with new infections.^14^

#### Implications of delayed recall

Screening policy is inevitably based on CIN3 diagnosis because of the extreme rarity of invasive cancer in screened women. The primary aim of screening is to prevent cancer by detecting and treating CIN3, but a major additional benefit is early cancer diagnosis. Most cervical cancers in regularly screened women are diagnosed at FIGO stage 1.^6^ The virtual absence of cancer in women aged under 20 years despite the large number of HPV infections from age 14 to 19 years^17^ suggests that a delay of 6–12 months in diagnosing CIN3 will not cause a detectable increase in cancer risk. Prevalent cancers, including all ten in ARTISTIC, almost always present with high‐grade cytology. In 8056 colposcopy referrals during the first round of the UK pilot, invasive cancer was diagnosed in two (0.035%) women following borderline/low‐grade cytology and in 56 (2.35%) following high‐grade cytology.^18^ This indicates that CIN3 is usually incipient following borderline/low‐grade cytology but is often prevalent in women with high‐grade cytology. The respective CIN3 and cancer risks beyond 2.5 years from entry to ARTISTIC were 3.3% (47/1407) and 0.4% (6/1407) in HPV+ women with normal cytology and 2.7% (23/852) and 0.1% (1/852) in HPV+ women with borderline/low‐grade cytology at entry (Table 1). A delay in retesting of a year for HPV16/18 and 2 years for other HPV types, might therefore be considered for borderline/low‐grade cytology. This would delay CIN3 diagnosis but will rarely delay cancer diagnosis. The minimal increase in the risk of developing cancer with a delay of 6 months to retesting of low grade cytology was accepted before HPV testing was introduced as a triage test for primary cytology screening. It is not clear that this risk–benefit balance should be abandoned for non‐16/18 HPV infection with low‐grade cytology, which is the normal manifestation of an active HPV infection, particularly in younger women.^19^

#### Colposcopy guidelines

Part of the continuing long‐term increase in cumulative incidence of CIN3+ in HPV+ women with normal cytology (Figure 1A) is due to occult CIN3 undetected by cytology. In a prospective study in the Netherlands six of the eight women with untreated CIN who regressed to normal cytology and colposcopy but remained HPV+ over several years had occult CIN3 detected by random biopsy.^20^ Current colposcopy guidelines^21^ do not recommend taking biopsies from women with normal, borderline or low‐grade cytology without an atypical transformation zone. This was based on the 8% CIN2+ risk in women attending for colposcopy but not eligible for biopsy whose HPV status was not known,^22^ which must be an underestimate for persistently HPV+ women. HPV+ women with normal cytology in the revealed arm of ARTISTIC were offered colposcopy if they were still HC2+ a year later. Among the 169 women who attended colposcopy, only half (81) of whom were biopsied, 9 CIN3s (5.3%) were diagnosed immediately and 15 CIN3s were diagnosed after further biopsies, a cumulative CIN3+ risk of 14.2%. It is not known how many more would have been diagnosed with random biopsy. A US trial reported an 8.5% prevalence of CIN3 in women with normal cytology referred to colposcopy and biopsy (directed or random) after a single positive test for HPV16/18.^23^ Women with HPV infection and normal cytology form the majority of the triage population, and there is an urgent need for better evidence to inform colposcopy management guidelines for these women.

#### Triage in older women

Among women aged <45 years who were HPV+ at entry, the long‐term CIN3 risk (16.0%) was unrelated to age but was lower (7.0%) in HPV+ women aged ≥45 years. These findings are similar to those reported from the UK pilot (4.8% in HPV+ women aged ≥50 years versus 9.1% in HPV+ women aged 30–49 years).^13^ This reduction could be due to physical difficulties in collecting adequate pathology^24^ or to under‐diagnosis of occult CIN3^20^ so a longer delay before retesting in older women might not be safe. Irrespective of cytology, postmenopausal women with persistent HPV might choose therapeutic excision of the transformation zone rather than repeated screening.

## Conclusions

Immediate referral of all HPV+ women with borderline or low‐grade cytology as recommended for the roll‐out of primary HPV screening will not be cost‐effective, particularly in women who have tested HPV‐negative in the previous round of screening. We suggest that low‐grade lesions can safely be retested to identify those with persistent HPV. Recall intervals of 1 year for HPV16/18 and 2 years for the remainder have been piloted in some regions for women with normal cytology and might also be considered for women with borderline/low‐grade cytology. The CIN3+ risk is confined to women with a persisting type‐specific HPV so partial genotyping test assays identifying HPV16/18 as a minimum provide more efficient risk stratification. The minimal risk of invasive cancer that has progressed beyond stage 1A must be weighed against the advantages for patients and the NHS of reducing the number of referrals to colposcopy.

The major unresolved issue, which will become increasingly important, is management of women with persistent HPV infection and normal cytology after two rounds of triage. A study should be conducted to evaluate whether random biopsy (or excision of the transformation zone in postmenopausal women) is substantially more sensitive than colposcopically directed biopsy for diagnosing disease in women with type‐specific HPV persistence and normal or low‐grade cytology.

### Availability of data

No additional data are available, in compliance with the ethical and governance regulations under which this research was undertaken.

### Disclosure of interests

CG received speaker fees from Roche paid to employer and a grant from NIHR‐HTA to fund this project. AS attended meetings with HPV assay manufacturers; speaker fees from Roche; travel and accommodation from Roche and Abbott for training and user group meetings; Roche, Abbott, Hologic, Becton Dickinson and Cepheid provided kits for assay validation purposes; PHE provided funding to support the NHS screening laboratory activity for the pilot. JP received a grant from NIHR‐HTA to fund this project. Completed disclosure of interest forms are available to view online as supporting information.

### Contribution to authorship

CG conducted all statistical analysis and drafted the report. AS conducted all HPV testing for the ARTISTIC trial. JP was the epidemiological PI of the ARTISTIC trial and contributed to the design of the trial, initiated the follow‐up study, and contributed to the statistical analysis and writing of the report.

### Details of ethics approval

Ethical approval of the study was obtained from the South East Coast NRES Committee (14/LO/0627) on 13 May 2014 and Section 251 support to process confidential patient information without consent was given by UK Health Research Authority Confidentiality Advisory Group.

### Funding

The work was funded by the NIHR‐HTA and Cancer Research UK.

## Supporting information


**Table S1** **.** Cumulative CIN3+ risks from round 2 by HPV partial genotype and cytology. Invasive cervical cancers (ICC) are also shown in brackets.
