# Supplementary material for: Triaging women with human papillomavirus infection and normal cytology or low‐grade dyskaryosis: evidence from 10‐year follow up of the ARTISTIC trial cohort
Source: BJOG. 2019 Nov 7;127(1):58–68. doi: 10.1111/1471-0528.15957 (PMC6916371; doi:10.1111/1471-0528.15957)
Supplement: Supplementary file 1 — Table S1 . Cumulative CIN3+ risks from round 2 by HPV partial genotype and cytology. Invasive cervical cancers (ICC) are also shown in brackets. [file BJO-127-58-s001.pdf]

Table S1. Cumulative CIN3+ risks from round 2 by HPV partial genotype and cytology. Invasive cervical cancers (ICC) are also shown in brackets

			Follow-up from round 2 (2.5 to 4 years after entry)								
**HPV/cytology at round 2**			**2.5 year risk from round 2**			**5 year risk from round 2**			**10 year risk from round 2**		
	**n (%[1]) at round 2**	**n CIN3+ (ICC)[2]**	**n CIN3+ (ICC)**	**%**	**95% CI**	**n CIN3+ (ICC)**	**%**	**95% CI**	**n CIN3+ (ICC)**	**%**	**95% CI**
**HR-HPV negative[3]**	12802 (94.7%)	21 (2)	11	0.09%	(0.05%, 0.15%)	15 (1)	0.12%	(0.07%, 0.19%)	21 (2)	0.17%	(0.11%, 0.26%)
**HR-HPV positive**	713 (5.3%)	60 (5)	51 (2)	7.2%	(5.5%, 9.3%)	55 (3)	7.7%	(6.0%, 9.9%)	60 (5)	8.4%	(6.6%, 10.7%)
**Normal cytology**											
**HPV16/HPV18**	151 (21.2%)	12 (2)	9 (1)	6.0%	(3.2%, 11.1%)	11 (1)	7.3%	(4.1%, 12.8%)	12 (2)	8.0%	(4.6%, 13.6%)
**Other HR-HPV**	333 (46.7%)	10 (3)	6 (1)	1.8%	(0.8%, 4.0%)	7 (2)	2.1%	(1.0%, 4.4%)	10 (3)	3.0%	(1.6%, 5.5%)
**All HR-HPV+**	484 (67.9%)	22 (5)	15 (2)	3.1%	(1.9%, 5.1%)	18 (3)	3.7%	(2.4%, 5.8%)	22 (5)	4.6%	(3.0%, 6.9%)
**Borderline/low-grade cytology**											
**HPV16/HPV18**	84 (11.8%)	13	13	15.5%	(9.3%, 25.2%)	13	15.5%	(9.3%, 25.2%)	13	15.5%	(9.3%, 25.2%)
**Other HR-HPV**	112 (15.7%)	9	7	6.3%	(3.0%, 12.7%)	8	7.1%	(3.6%, 13.8%)	9	8.1%	(4.3%, 14.9%)
**All HR-HPV+**	196 (27.5%)	22	20	10.2%	(6.7%, 15.4%)	21	10.7%	(7.1%, 16.0%)	22	11.2%	(7.5%, 16.6%)
**Moderate/Severe cytology**											
**HPV16/HPV18**	20 (2.8%)	8	8	40.0%	(22.4%, 64.3%)	8	40.0%	(22.4%, 64.3%)	8	40.0%	(22.4%, 64.3%)
**Other HR-HPV**	13 (1.8%)	8	8	61.5%	(37.2%, 86.0%)	8	61.5%	(37.2%, 86.0%)	8	61.5%	(37.2%, 86.0%)
**All HR-HPV+**	33 (4.6%)	16	16	48.5%	(33.2%, 66.5%)	16	48.5%	(33.2%, 66.5%)	16	48.5%	(33.2%, 66.5%)
**All women from round 2**	13515[4]	81 (7)	62 (2)			70 (4)			81 (7)		

[1]Percentages by cytology are given out of total 680 HR-HPV+ women
[2]In 12 to 14 years of follow-up
[3]HC2 negative or HC2 positive with no HR-HPV detected
[4]76 women with no cytology taken at round 2 are excluded from the table
